# Stakeholder perspectives of tobacco use on campus and implementation of a tobacco-free policy at a Midwest university

**DOI:** 10.18332/tpc/199932

**Published:** 2025-02-10

**Authors:** Olufunmilola Abraham, McKennah J. Matulle, Jenny S. Li, Sydney Thao, Ellie Maday, Qianqian Zhao

**Affiliations:** 1 Department of Pharmacy Practice and Science, College of Pharmacy, University of Kentucky, Lexington, United States; 2 School of Pharmacy, University of Wisconsin-Madison, Madison, United States; 3 Department of Biostatistics and Medical Informatics, University of Wisconsin-Madison, Madison, United States

**Keywords:** tobacco policy, survey, college campus, secondhand aerosols

## Abstract

**INTRODUCTION:**

Implementation of a 100% tobacco-free policy at universities can assist in limiting the potential negative health impacts of tobacco use, such as susceptibility to lung and heart disease, cancer, addiction, and life-long use. This study’s goal was to gain the perspective of students and non-students across a large Midwestern university campus on implementation of a 100% tobacco-free policy.

**METHODS:**

Students, faculty, and staff of a Midwestern university were recruited to complete a 19-question cross-sectional online survey on tobacco use on campus, awareness of the current tobacco-free policy, and their interest in supporting a 100% smoke-free policy on campus. The survey included open- and close-ended questions, and responses were analyzed qualitatively and quantitatively.

**RESULTS:**

A total of 2389 respondents completed the survey, and 291 (12.2%) reported current tobacco use from April to July 2024. Participants with a higher probability of current tobacco use were associated with having a higher degree of exposure to secondhand aerosols (AOR=1.34; 95% CI: 1.10–1.62), more awareness of the current tobacco policy (AOR=1.19; 95% CI: 1.06–1.32), and disagreed with the petition statement in support of a 100% tobacco-free campus policy (AOR=2.47; 95% CI: 1.48–4.12). Participants that reported a higher degree of exposure to secondhand aerosols (AOR=2.18; 95% CI: 1.19–3.99) and agreed with the statement that a 100% smoke-free campus policy would promote a healthier college campus (AOR=2.18; 95% CI: 1.20–3.96) were significantly associated with supporting the petition for a 100% smoke-free policy on this university campus.

**CONCLUSIONS:**

Supporting a 100% tobacco-free policy for a healthier and safer university campus was demonstrated to be associated with secondhand aerosol exposure among survey respondents.

## INTRODUCTION

Tobacco use is the number one risk factor for many diseases, from lung and heart disease to many cancers^1^. Smoking continues to be the leading cause of preventable deaths, with 1 in 5 deaths associated with cigarette use^1^. Despite more than 50 years of research confirming the deleterious health risks and consequences of tobacco use, Americans continue to smoke-tobacco and nicotine-containing products, such as electronic cigarettes (e-cigarettes)^1,2^. Additionally, there is strong evidence of the dangerous health effects of secondhand smoke and urgent concern on the potentially harmful effects of secondhand e-cigarette aerosols^1,3^. Across the nation, there are 46 million tobacco users, and 17% of college-aged young adults (18–24 years), are current users^4^. This young adult age group is also the most likely to use e-cigarettes compared to all other adult age groups, with 11% of participants aged 18–24 years regularly vaping^2^.

Young adults who use tobacco are particularly vulnerable and at risk for lifelong tobacco addiction^5^. Almost 90% of current smokers started before the age of 18 years, and 98% started smoking before the age of 26 years^1^R. As the brain does not reach full development until the age of 25 years, nicotine use by those aged 18–24 years continues to make detrimental changes to their brain functionality, contributing to addiction susceptibility^1,6^. Young adult tobacco users are not only at risk for tobacco addiction but also other substance use disorders^7^. One way that has been proven to control and decrease tobacco use is through policies, such as 100% tobacco-free location policies^8,9^. With almost 40% of young adults aged 18–24 years enrolled in 2- or 4-year college institutions^10^, there is an urgent need for implementation of tobacco-free policies on these campuses to reduce and prevent tobacco use among college students^11^.

The definition of a ‘100% tobacco-free’ campus policy is that use of any tobacco or nicotine-containing product (cigarettes, e-cigarettes, smokeless tobacco, etc.) is prohibited on the entire campus property, both indoors and outdoors, including parking lots, stadiums, residential housing, and any other locations where people may use these products^11^. For the purpose of this study, the term ‘tobacco product’ will be used as an umbrella term to include both tobacco and nicotine-containing products. Some exemptions for tobacco-free campuses may include personal vehicles, scientific studies on tobacco, or traditional Indigenous spiritual or cultural ceremonies^11^. Tobacco-free campus policies create an environment that limits secondhand smoke exposure, discourages tobacco use, helps prevent initiation of tobacco use, and potentially increases tobacco cessation^1,11^.

At the large, Midwestern university where this study took place, the current tobacco policy was implemented in 2016 and contains many limitations^12^. This policy states that smoking or vaping of any substance is prohibited in all indoor spaces (buildings, facilities, vehicles) owned, operated, or leased by the university, except in smoking-allowed university housing, as a part of a theatrical performance, and the other permissible exceptions listed earlier^12^. Some key limitations of this policy are that smokeless tobacco remains permissible indoors, smoking and vaping outdoors on campus is allowed, and the exceptions of smoking-allowed campus housing and theater performances with smoking. Currently, only the university’s Health Sciences facilities and grounds, the university’s Arboretum, and the student unions remain smoke-free both indoors and outdoors^12^.

A previous survey of college students at this university found that a third of undergraduate students and 1 in 7 graduate and professional students have used tobacco at least once^13^. Students, faculty, and staff are all at risk of exposure to secondhand smoke and secondhand aerosols on campus. As of April 2024, there were more than 2617 campus sites that were 100% smoke-free in the United States and, yet, some large universities maintain a limited tobacco policy^14^. Universities that have adopted smoke-free campus policies have observed a decrease in tobacco use^15^. Additionally, these universities have seen changes in their campus communities’ perceptions and opinions of tobacco use since adding smoke-free policies^15^.

In recent years, different organizations, such as the American Lung Association and Public Health department, have worked towards building and implementing a 100% tobacco-free policy. This study conducted an online survey among students, faculty, and staff to better understand current tobacco use on campus, perspectives on the current university tobacco policy, and assess the potential support for a stronger tobacco-free policy.

## METHODS

### Study design and procedures

From April to July 2024, all students, faculty, and staff at this large, American university were invited to complete an online cross-sectional survey on their tobacco use and perceptions as well as their perspective on a 100% tobacco-free campus-wide policy. The online survey was distributed via a university-wide mass email listserv to 44238 individuals affiliated with the university as well through paper flyers distributed across campus. This study defined affiliation with the university as having some form of connection with the university, either as a student or employee of the institution. Additionally, two tobacco awareness events led by health sciences organizations from January through May 2024 took place to provide attendees an opportunity to complete the survey. A total of 39 people attended these two events. All survey respondents from both the email distribution and these two events were entered into a raffle to win one of many prizes, which were university-branded gifts. Raffle prize winners were randomly chosen through an online randomization generator and notified via email to collect their prize after closing of the online survey and before analysis began.

### Survey development

The survey was adapted from an unvalidated and unpublished survey developed by epidemiologists at Public Health Madison & Dane County. A few additional questions were added by the investigators to elicit additional open-ended responses from survey respondents. Questions focused on university-affiliated demographics, tobacco use, perceptions on tobacco use on campus, awareness of the current university tobacco policy, and support for a change in the current tobacco policy. There were 16 close-ended questions and 2 open-ended questions, which can be seen in Supplemental file Material 1. There was an additional question asked regarding interest in actively participating with the research team to pursue tobacco policy change, but this question was not analyzed for this study.

### University-affiliated demographics

The first questions asked about university-affiliated demographics, such as how the respondent was affiliated with the university, the length of time of their affiliation with the university, and where they resided for the current year. On how the respondent was affiliated with the university, options included being an undergraduate student, graduate student, non-degree student (university special student, guest auditor, or noncredit continuing education student), faculty/staff/employee, other (with write-in option), or not affiliated with the university. Those who selected ‘not affiliated with the university’ were unable to complete the survey. For university-affiliation length of time, respondents were able to type in the number of years that they had been affiliated with the university. This measure also specified that respondents could combine their years of affiliation, if they were once a student and currently an employee, or vice versa. When asking about residence in the current year, respondents chose between university housing (which was defined as living in a residence hall, residential learning community, or university apartment), fraternity or sorority housing, off-campus residence (house, apartment, condo, etc.), or other (with a write-in option).

### Tobacco use

Regarding current tobacco use, one of the study’s primary outcomes, respondents were asked if they had used any tobacco product or electronic vaping devices in the past 30 days, with ‘yes’ or ‘no’ as response options. Respondents who responded ‘yes’ to the tobacco use question would be asked additional questions about their tobacco use, such as what products they used in the past 30 days, when did they try their first tobacco product, and to describe the frequency of their tobacco use during their time at the university. For tobacco products used in the past 30 days, respondents were allowed to check all that applied from a list with the following options: cigarettes; other tobacco products such as cigars, little cigars, cigarillos, pipes, or hookah; smokeless tobacco products such as chewing tobacco, snuff, snus, dip, orbs, sticks, or strips; electronic vaping products such as e-cigarettes, vape pens, hookah pens, or e-pipes; oral nicotine pouches; or other (with a write-in option). For the question on at what age respondents first tried a tobacco product, the options were: <18, 18–20, ≥21 years, or other (with a write-in option). Regarding frequency of tobacco use during their time at the university, respondents were asked to select between the options: increased, remained the same, or decreased.

### Perceptions on tobacco use on campus

Questions on respondent perceptions of current tobacco use on campus focused primarily on secondhand smoke and secondhand vape aerosols and their exposure to these on campus. Secondhand smoke was defined as ‘smoke that you may breathe in that comes from someone else using a tobacco product’. Respondents were asked how harmful they believed secondhand smoke is to one’s health, with 5-point Likert scale response options of: 1) not at all, 2) a little, 3) somewhat, 4) very, and 5) extremely . They were also asked how often they were exposed to secondhand smoke while they are on the university’s campus, with 5-point Likert scale response options of: 1) never, 2) sometimes, 3) about half the time, 4) most of the time, and 5) always . Secondhand aerosols from vaping were defined as aerosols you may breathe in that come from someone else using an electronic vaping device (e-cigarette, vape pen, etc.). The same questions regarding secondhand smoke were asked about secondhand vape aerosols, with the same response options.

### Awareness of the current tobacco policy and support for tobacco policy change

On awareness of the current university tobacco policy, respondents were asked to select from 5-point Likert scale response options of: 1) very unaware, 2) somewhat unaware, 3) neither aware nor unaware, 4) somewhat aware, and 5) strongly aware. On the following page of the online survey, separate from this awareness question, the survey described the limited locations on campus where there are 100% tobacco-free zones, which consist of the health sciences campus, the university arboretum, facilities owned by the family medicine department, and one of the student union buildings and facilities. There is also an external link provided for those interested in reading the full text of the current tobacco policy on campus. There are then two questions asking respondents on how much they agree or disagree with statements related to the potential impact of a 100% tobacco-free campus policy. The first statement is: ‘A 100% tobacco- and vape-free campus policy at the university would encourage tobacco users to quit or reduce their consumption’; the second statement is: ‘A 100% tobacco- and vape-free policy at the university would promote a cleaner, greener, and healthier campus’. For each statement, respondents were asked to select how much they agreed or disagreed with the statement, using a 5-point Likert scale of: 1) strongly disagree, 2) somewhat disagree, 3) neither agree nor disagree, 4) somewhat agree, and 5) strongly agree . Another 5-point Likert scale question was asked to respondents regarding how much they would support or oppose a 100% tobacco-free campus policy for the entire university campus. The response options were that they would: 1) strongly oppose, 2) somewhat oppose, 3) neither oppose nor support, 4) somewhat support, and 5) strongly support. As one of our primary outcomes, the last close-ended question in the survey was a final petition statement and asked if respondents agreed, disagreed, or preferred not to answer. The petition statement was: ‘I support the restructuring of the university campus policy on tobacco to be 100% tobacco-free (no smoking, vaping, nor smokeless tobacco use) on all indoor and outdoor grounds’. The last two questions on the survey were both open-ended, with the first one asking respondents who agreed with the petition statement to provide a statement of why they support the restructuring of the university tobacco policy. The second open-ended question asked respondents if they had any other comments or questions regarding the university tobacco policy.

### Statistical analysis

Statistical analyses were conducted using SAS software (SAS Institute Inc., Cary NC), version 9.4. All reported p-values were two-sided and p<0.050 was used to define statistical significance. Descriptive statistics such as frequency and percentage were generated for categorical variables and mean and standard deviation (SD) were generated for continuous variables. Fisher’s exact test and t-test were used for comparing tobacco users and non-users, also for petition supporters versus non-supporters for univariate comparisons. Multivariable logistic regression model with stepwise forward model selection method was used to find what variables were associated with current tobacco use; and among current tobacco users, which were associated with support for petition of tobacco policy change. The stepwise selection method chose which variables to keep in the model, resulting in adjusted analyses. Adjusted odd ratios (AORs) and 95% confidence intervals (CI) were calculated as well.

The addition of the two open-ended survey questions were designed to address the participant’s reasoning for supporting the restructuring of the campus tobacco policy and aid in distinguishing areas of concern in presenting a new campus-wide tobacco policy. An inductive thematic analysis was conducted by the research team members to analyze survey responses to the open-ended questions. In this process, survey responses to each individual question were read to understand the subject matter and group the commentary accordingly. Next, responses with similar ideas were grouped together to formulate an overarching theme for data analysis. The predominant themes that emerged from the data collected were discussed amongst research members to reach a consensus and address any differences that arose.

## RESULTS

### Respondent demographics

A total of 2538 university stakeholders began the survey, yet 2389 respondents completed the survey. For the analysis of this study, all respondents who completed the full survey (n=2389) were included in the analysis. Of the respondents, 40.6% (n=969) were university students, and 59.4% (n=1420) were nonstudents at the university (i.e. faculty, staff, employees, etc.). Most respondents lived off-campus (90.5%, n=2154), and 41.2% (n=978) were at the university for over five years. Demographic information can be found in Table 1.

**Table 1:** University-affiliated demographics of survey respondents. Results from a cross-sectional survey of university stakeholders (students and employees) on perspectives campus-wide tobacco use and the campus tobacco policy, 2024 (N=2389)

*Characteristics*	*Current user (N=291) n (%)*	*Non-user (N=2090) n (%)*	*All (N=2389) n (%)*	*p*
**Student**				<0.0001
Yes	189 (65)	780 (37)	969 (41)	
No	102 (35)	1310 (63)	1420 (59)	
**Affiliation with the University in years**				<0.0001
0–1	114 (39)	624 (30)	738 (31)	
2–5	114 (39)	545 (26)	659 (28)	
>5	62 (21)	916 (44)	978 (41)	
**Live on Campus**				0.019
Yes	39 (13)	187 (9)	226 (10)	
No	252 (87)	1902 (91)	2154 (90)	

Fisher’s exact test was performed to derive the p-values. P-values are unadjusted.

### Descriptive findings on current tobacco users

Regarding current tobacco use, 12.2% (n=291) of respondents reported use of tobacco products within the last 30 days. Of those current tobacco users, 55.2% (n=160) tried their first tobacco product before the age of 18 years, 32.8% (n=95) tried their first tobacco product at 18–20 years, and 10% (n=29) tried their first tobacco product after the age of 21 years. The most used tobacco products reported by current tobacco users were cigarettes (45.36%, n=132) and electronic vaping products (60.82%, n=177). Of the 291 current tobacco users who answered this question on the survey, more respondents reported having increased their use of tobacco products while affiliated with this university (44.6%, n=129) compared to those who felt it remained the same (33.9%, n=98) or decreased (21.5%, n=62) during their time on the campus.

Table 2 shows the adjusted odds ratio of being a current tobacco user with university affiliation and number of years at the university. University students had lower odds of identifying as a current tobacco user compared to faculty and staff at the university (AOR=0.53; 95% CI: 0.37–0.74, p<0.001). Years affiliated with this university’s campus (0–1 year vs 2–5 years vs ≥5 years) were significantly associated with current tobacco use (p=0.007). Participants with over 5 years of campus affiliation had lower odds of using tobacco-containing products when compared to those with 0–1 year campus affiliation (AOR=0.60; 95% CI: 0.39–0.91, p=0.017). Participants affiliated with the campus for 2–5 years were not significantly different than those with 0–1 year affiliation (AOR=1.18; 95% CI: 0.85–1.63, p=0.333).

**Table 2:** Multivariable logistic regression model results of association of current tobacco users with university-affiliated demographics and key survey items. Results from a cross-sectional survey of university stakeholders (students and employees) on perspectives campus-wide tobacco use and the campus tobacco policy, 2024 (N=2389)

*Variables*	*Current tobacco users*		
	*AOR*	*95% CI*	*p*
**University stakeholder** (compared with students)			
Non-students	0.53	0.37–0.74	<0.001
**Years affiliated with the university** (compared with <2 years)			
2–5	1.18	0.85–1.63	0.333
>5	0.60	0.39–0.91	0.013
Perceived secondhand smoke as harmful	0.69	0.59–0.81	<0.001
Exposed to secondhand e-cigarette aerosols on campus	1.34	1.10–1.62	0.003
Perceived a 100% tobacco-free campus policy would positively impact health and the environment on campus	0.83	0.71–0.97	0.019
Aware of the current campus tobacco policy	1.19	1.06–1.32	0.002
Support for a 100% tobacco-free campus policy	0.64	0.54–0.78	<0.001
Opposed a 100% tobacco-free campus policy	2.47	1.48–4.12	0.001

AOR: adjusted odds ratio; adjusting for the remaining variables in the table.

### Comparison of all survey respondents and current tobacco users on tobacco harm perceptions

Regarding secondhand smoke, more than 96% of all respondents perceived secondhand smoke to be either very harmful (62.4%, n=1486) or somewhat harmful (33.3%, n=792) to one’s health. However, the perception that secondhand smoke was more harmful had lower odds of being perceived by current tobacco users (AOR=0.69; 95% CI: 0.59–0.81, p<0.001)(Table 2 and Supplementary file Material 2). Regarding exposure to secondhand e-cigarette aerosols on campus, 34.1% were never exposed to vaping (n=812), 55.1% were sometimes exposed (n=1309), and only 7.8% were exposed about half their time on campus or more (n=185). There was a significantly higher perceived exposure to secondhand aerosols amongst current tobacco users compared to non-users (89% vs 71%, respectively; p=0.014) (Table 3). The perceived higher exposure to secondhand e-cigarette aerosols had higher odds of being from a current tobacco user (AOR=1.34; 95% CI: 1.10–1.62, p=0.003). Most respondents either strongly agreed (52.7%, n=1254) or somewhat agreed (31.4%, n=748) with the following statement on the health and environmental impact of a stronger tobacco policy: ‘A 100% tobacco- and vape-free policy would promote a cleaner, greener, and healthier campus’. However, a higher degree of agreement with the statement had lower odds of being from a current tobacco user (AOR=0.83; 95% CI: 0.71–0.97, p=0.013) (Table 2).

**Table 3:** Perceptions of tobacco use amongst survey responses. Results from a cross-sectional survey of university stakeholders (students and employees) on perspectives campus-wide tobacco use and the campus tobacco policy, 2024 (N=2389)

*Perceptions*	*Current user (N=291) Mean (SD)*	*Non-user (N=2090) Mean (SD)*	*All (N=2389) Mean (SD)*	*p*
Perceived secondhand smoke (SHS) as harmful	4.14 (0.78)	4.63 (0.58)	4.57 (0.63)	<0.001
Degree of exposure to SHS	1.67 (0.67)	1.73 (0.61)	1.72 (0.62)	0.154
Perceived secondhand aerosols (SHA) as harmful	3.53 (1.02)	4.19 (0.80)	4.11 (0.86)	<0.001
Degree of exposure to SHA	1.92 (0.89)	1.79 (0.71)	1.80 (0.73)	0.014
Awareness of current tobacco-free policy	2.86 (1.41)	2.81 (1.31)	2.81 (1.32)	0.561
Agreement on 100% tobacco-free policy will reduce consumption	2.50 (1.36)	3.46 (1.22)	3.34 (1.27)	<0.001
Agreement of 100% tobacco-free policy promotes a healthy campus	3.29 (1.35)	4.37 (0.93)	4.24 (1.05)	<0.001
Degree of support for tobacco-free policy	2.90 (1.43)	4.38 (1.00)	4.20 (1.65)	<0.001

Results are scored on a 5-point Likert scale. T-test was performed to derive the p-values. P-values are unadjusted.

### Varying attitudes towards implementation of a 100% tobacco-free campus policy

Respondents had a wide range of awareness regarding the current campus tobacco policy, with 45.3% (n=1079) reporting as either somewhat or very unaware of the policy, 17.2% (n=409) were unsure of their awareness, and 37.5% (n=893) were either somewhat or very aware of the policy. In terms of awareness of the university’s current tobacco policy, the higher awareness of the current tobacco policy had higher odds of being of a current tobacco user (AOR=1.19; 95% CI: 1.06–1.32, p=0.002) (Table 2 and Supplementary file Material 2).

Most respondents (79.0%, n=1882) either somewhat or strongly supported the implementation of a 100% tobacco-free campus policy. With the final petition statement ‘I support the restructuring of the campus policy on tobacco to be 100% smoke/vape-free on all grounds’, 71.1% (n=1693) of respondents agreed, 15.9% (n=381) disagreed, and 12.9% (n=307) chose ‘prefer not to answer’. Most non-tobacco users (n=2090) agreed with the petition statement (76.4%, n=1597), and most tobacco users (n=291) disagreed (52.9%, n=154). Of the current tobacco users who agreed with the petition statement (33%, n=96), 74% (n=71) of respondents identified as students, and 42% (n=40) of respondents were affiliated with the university for less than 1 year (Table 4). The perceived higher exposure to secondhand e-cigarette aerosols had higher odds of being from a supporter of a 100% smoke-free campus policy (AOR=2.18; 95% CI: 1.19–3.99, p=0.012) (Table 5 and Supplemental file Material 3). However, opposing the petition statement had higher odds of being a current tobacco user (AOR=2.47; 95% CI: 1.48–4.12, p<0.001).

**Table 4:** Petition support amongst current tobacco users. Results from a cross-sectional survey of university stakeholders (students and employees) on perspectives campus-wide tobacco use and the campus tobacco policy, 2024 (N=2389)

*Characteristics*	*Current tobacco users*		
	*Agree with petition (N=96) n (%)*	*Disagree with petition (N=154) n (%)*	*p*
**Student**			0.015
Yes	71 (74)	90 (58)	
No	25 (26)	64 (42)	
**Year of affiliation with the university**			0.544
0–1	40 (42)	55 (36)	
2–5	39 (41)	63 (41)	
>5	17 (18)	35 (23)	
**Stakeholders living on campus**			1.000
Yes	12 (13)	20 (13)	
No	84 (87)	134 (87)	
**Tobacco use status**			
Cigarette consumption	32 (33)	77 (50)	0.013
Use of other smoked tobacco products	7 (7)	25 (16)	0.051
Smokeless tobacco use	2 (2)	10 (6)	0.137
Vaping product use	61 (64)	86 (56)	0.238
Oral nicotine use	17 (18)	32 (21)	0.625
**Frequency change of tobacco product use**			0.056
Increased	46 (48)	51 (40)	
Stayed the same	25 (26)	63 (41)	
Decreased	24 (25)	29 (19)	
***Perceptions***	***Mean (SD)***	***Mean (SD)***	
Perceived secondhand smoke (SHS) as harmful	4.35 (0.70)	3.99 (0.84)	<0.001
Degree of exposure to SHS	1.77 (0.75)	1.56 (0.56)	0.018
Perceived secondhand aerosols (SHA) as harmful	3.83 (1.00)	3.32 (1.01)	<0.001
Degree of exposure to SHA	2.08 (0.99)	1.76 (0.77)	0.007
Awareness of current tobacco-free policy	2.78 (1.36)	2.86 (1.43)	0.679
Agreement on 100% tobacco-free policy will reduce consumption	3.51 (1.29)	1.86 (1.05)	<0.001
Agreement of 100% tobacco-free policy promotes a healthy campus	4.41 (0.78)	2.57 (1.22)	<0.001
Degree of support for tobacco-free policy	4.34 (0.81)	1.90 (0.97)	<0.001

Fisher’s exact test was performed to derive p-values for categorical variables. T-test was used to derive p-values for perception scales (1–5). P-values are unadjusted.

**Table 5:** Multivariable logistic regression model results of association of those agreeing to a 100% tobacco-free campus policy with key survey items. Results from a cross-sectional survey of university stakeholders (students and employees) on perspectives campus-wide tobacco use and the campus tobacco policy, 2024 (N=2389)

*Variables*	*Agreed with petition for a 100% tobacco-free campus policy*		
	*AOR*	*95% CI*	*p*
Exposed to secondhand smoke on campus	2.180	1.191–3.991	0.012
Agreement on tobacco products affecting one’s health	2.176	1.196–3.9661	0.011
Support of tobacco-free campus policy	6.825	3.729–12.493	<0.001

AOR: adjusted odds ratio; adjusting for the remaining variables in the table.

### Thematic analysis of restructuring the university’s tobacco-free policy

Participants provided justification for their support of a tobacco-free college campus. A total of 1251 participants (52.4%) explained their reasoning for supporting the tobacco-free policy. Five responses were not included due to lack of response to the research question. Three major themes analyzed from these data included participant support for the betterment of public health, the limitation of secondhand smoke exposure, and the control of the harmful effects of vaping. Supplementary file Material 4 contains example quotes from respondents providing their reasoning for support of a 100% tobacco-free policy.

Most participants who disclosed their reasoning for support of a 100% tobacco-free campus policy described the destruction caused by tobacco-containing products. One participant described the detriment tobacco poses to their health: ‘I don’t think that tobacco is good for our health, and the health of others around us non-tobacco user’. In another accord, the age to which individuals are exposed to products with this proposed harm was expressed: ‘Tobacco use is incredibly harmful, especially to those who are too young to fully understand how their decision affects their long-term health’.

In terms of public health, a predominant reasoning for support of a 100% tobacco-free policy centered around the negative effects on the environment. Another non-tobacco using participant believed: ‘Tobacco and vaping are harmful to both health and the environment’, with another respondent adding with regard to the environmental impact tobacco waste produced: ‘[A tobacco-free campus policy] promotes clean air and a clean campus (through reduced disposal of cigarette butts on the ground)’. Interestingly, one current tobacco user indicated their support for a 100% tobacco-free policy because: ‘It will create a positive and cleaner environment to everyone’.

Regarding secondhand smoke exposure, some responders indicated the effect tobacco-containing products pose to bystanders on campus. One response noted: ‘People do not have a choice to avoid secondhand smoke’, adding that ‘Younger people may be exposed to secondhand smoke from friends and not feel comfortable avoiding it’, who represent a common population seen on college campuses. A second participant who provided the unique perspective as a former smoker added: ‘I think that it is important to actually be thoughtful to those who do not smoke, I also see many smokers that ‘hide’ so they can smoke around the health science campus’.

Furthermore, 744 participants provided comments or questions about the current campus tobacco policy. Supplementary file Material 5 contains the participants’ comments and questions on the reconstruction of the current tobacco policy at this university. Three major themes appeared as participants provided their observations on the tobacco-policy restructuring, including tobacco cessation efforts, tobacco-free policy enforcement, and tobacco-free policy implementation.

One major theme of the participants’ responses was centered around ensuring assistance to those quitting the use of tobacco-containing products, if tobacco use was limited on campus. One non-tobacco using respondent noted: ‘Are there resources free to students in assisting them in quitting if they are interested?’. A current tobacco user who described their tobacco-product use as increased whilst on campus added: ‘Along with new policies, I think introducing new quitting resources and ad campaigns about quitting would be more beneficial’.

Additionally, participants questioned the implementation process of new policies and how they should act when encountering tobacco-free policies. Two examples from non-tobacco using respondents are: ‘How would this [new tobacco policy] be enforced if someone [was] to be seen using tobacco products?’ and ‘How much education, time, and resources will be committed to sharing the policy?’. One final theme predicated the limitations in a 100% tobacco-free campus, such as one comment by a current tobacco user: ‘Having a “smoke-free” campus is extremely difficult to enforce, both indoors and outdoors’. A non-tobacco using respondent shared that ‘While I fully support the policy in theory, there isn’t much point in a policy if people don’t or can’t follow it’.

## DISCUSSION

This study examined perceptions of the current tobacco policy at this large, Midwestern university’s campus. Current tobacco users and non-tobacco users were found to have significant differences in key factors related to the overall impact of tobacco products on university stakeholders and support for a 100% tobacco-free campus policy at this university.

Most current tobacco users in this study either increased or maintained their use of tobacco-containing products during their time affiliated with the campus. According to the Centers for Disease Control (CDC), young adults (18–24 years) report an increased use of electronic cigarettes when compared to other age groups^16^, which is aligned with this study’s findings. Furthermore, the CDC published that cigarettes and electronic cigarettes are the most common tobacco products used by adults^4^. These were also the most used tobacco products by study respondents, which are both threatening to students on college campuses with their increased prevalence amongst this young adult population.

Our study found that university students showed similar tobacco use when compared to faculty and staff. These findings imply that tobacco may be on the rise among young adults and negatively influence even younger populations who visit the campus. This study further provides insight into both the student, faculty, and staff perspective on current tobacco use on campus and the perceived health policy implications, which has been absent from prior studies researching the perceptions of students on college campuses.

These findings allow us to better understand the status of secondhand smoke and e-cigarette aerosols on campus and the perceptions of university students and staff. One way the university’s current tobacco policy limits secondhand smoke is by enforcing a 100% smoke-free zone within the Health Science’s campus. This element to the campus policy provides protection against secondhand smoke and e-cigarette aerosols to those in this zone. While this area provides a safety net against the harm of secondhand smoke and e-cigarette aerosols, the rest of campus continues to be at risk. After implementation of a 100% tobacco-free campus policy, a study by Nyman et al.^17^ found that participants were less likely to encounter secondhand smoke on campus. A 100% tobacco-free policy on this university’s campus could generate similar results for the campus population if given the opportunity for enforcing a stronger tobacco policy in the future.

Current tobacco users in this study demonstrated higher awareness of the current campus tobacco policy and more opposition to a 100% tobacco-free policy. These results have implications for understanding the correlation between the use of tobacco products and the perceptions of the current tobacco policy. Current tobacco users know the existing restrictions on their tobacco use and would oppose a stricter policy that limits the use of tobacco products. This demonstrates the need for educational and behavioral interventions for current tobacco users on campus to encourage tobacco cessation and desire for a healthier lifestyle.

Participants in support of a 100% smoke-free campus policy alluded to the harmful nature of products containing tobacco, the promotion of a cleaner campus environment, and the protection against secondhand smoke that the policy could provide. A similar study conducted amongst young adults concluded with a similar perception that tobacco-containing products along with marijuana products were harmful to their users due to the effects of the chemicals at high concentration within the body^18^. Aside from damages posed to one’s health, damages to the environment of the campus preceded the notion for this campus policy support. An initiative at two San Diego college campuses found and collected 23885 and 6525 cigarette butts, at each respective campus, which signified the waste that tobacco products present to the environment^19^. Next to the environmental impact, secondhand smoke to those who choose not to use tobacco products creates an additional hazard to their health. According to the CDC, secondhand smoke exposure rates have decreased within the United States, which could in part be due to limitations of smoke-free policies across public places^20^. Initiatives like those proposed on this university’s campus could help assist with these endeavors for this vulnerable population.

Given the opportunity for commentary on tobacco-free campus policies across this university’s campus, implementation and enforcement of a 100% tobacco-free campus policy can be an area of concern for some. A similar survey presented to students at an urban university proposed a similar issue of enforcement of a tobacco-free policy due to the large campus premises and inability to reprimand all persons in violation of the new policy^21^. Suggested evidence-based strategies have been proposed to implement a tobacco-free policy that could potentially curb the limitations to imposing such a policy^22,23^.

Participants, independent of their support for a 100% tobacco-free policy, did question availability of assistance for those on campus who were looking to quit use of tobacco products. Those in support of a 100% tobacco-free policy recommended a secondary approach to limiting tobacco usage on college campuses whilst also helping them refrain from these products. One proposed mechanism to unite students with resources is through the implementation of training programs within the student health services^24^. Some current tobacco users desired to know how to quit and university student services can provide the opportunity for such programs to address this concern of campus participants. Further research endeavors can investigate the utility of quitting resources for current tobacco users on university campuses.

### Implications

Although current tobacco users had higher odds of opposing a 100% tobacco-free policy at the university, more than 70% of survey respondents supported a stronger policy. Findings from this study demonstrated that the university stakeholders who support a stronger policy perceive that it would make the campus healthier. However, there would be a strong need to still address the concerns of the current tobacco users and those who oppose the stronger tobacco policy. The criteria for implementation and enforcement of this policy need to be further established to properly address the concerns of users of tobacco-containing products. These results make an important distinction between those who support and oppose a 100% tobacco-free policy, and both groups should be supported in the revised tobacco policy for this university’s campus.

### Strengths and limitations

One strength of this study is the large sample size with representation from the campus’ students, faculty, and staff, enhancing the survey results to properly represent those who visit the campus. Secondly, the survey received a high response rate among those completing the survey which allowed for the level of data analysis achieved within this study. Finally, this study provided relevant insights into tobacco policies on college campuses which can be utilized to further improve current practices on campus to promote a healthier lifestyle for those associated with the university’s campus.

This study does have its limitations. The survey instrument used in this study was not validated for internal consistency prior to use, so future studies utilizing this instrument will assess the validity and reliability of the instrument to measure what it intends to measure. Additionally, no power analysis was completed prior to data collection for this study. However, future survey studies with this instrument will conduct a power analysis to assess the ideal sample size of participants for optimal results. The online survey did not collect demographic information, such as age, gender, education level, or ethnicity, limiting the ability to compare the findings across these demographics as well as generalize the results for future campus policy changes across other countries. The absence of demographic data in this study also meant that the survey data could not be weighted, nor compared to the demographics of the entire university population. Future iterations of this university-wide survey will include collection of demographic data to address these limitations. Also, the cross-sectional design of the survey limits the ability to assess how the results change over time and response bias can be introduced during a survey design. Due to the recruitment of survey respondents through university engagement and the potential stigma surrounding tobacco use amongst campus population, participants can provide responses that are more socially acceptable or expected, which could contribute to social acceptability bias and alter the study’s results. Though our observational study questioned respondents’ exposure to secondhand smoke and aerosols, with the nature of these products, the unknown secondhand exposure to tobacco containing products is difficult to quantify. Additionally, there was a small group of respondents who completed the survey as a part of the health awareness activities, which may have made them more likely to express support for the policy. The number of respondents from this group was not measured nor identified, so these responses would not be able to be removed from analysis.

## CONCLUSIONS

This study’s findings demonstrate how current tobacco use could impact college campus tobacco policy perceptions. Implementation of a 100% tobacco-free college campus policy is one way these dangerous products can be limited in this vulnerable population. Current tobacco users oppose a stronger tobacco policy, as it would further limit their tobacco use on campus. These findings can support and guide the development and implementation of a 100% tobacco-free policy on the university’s campus, updating the current policy implemented in 2016. Future studies should analyze demographic variabilities among current tobacco users and non-tobacco user’s perception of a 100% tobacco-free policy, as well as dive deeper into development of supportive programs for current tobacco users looking to quit using tobacco products. Further evidence is needed to examine the long-term effects and implications of a 100% tobacco-free policy on this university’s college campus.

## Supplementary Material



## Data Availability

The data supporting this research are available from the authors on reasonable request.
