# Supplementary material for: Stakeholder perspectives of tobacco use on campus and implementation of a tobacco-free policy at a Midwest university
Source: Tob Prev Cessat. 2025 Feb 10;11:10.18332/tpc/199932. doi: 10.18332/tpc/199932 (PMC11808339; doi:10.18332/tpc/199932)
Supplement: Supplementary file 1 [file TPC-11-12-s1.pdf]

Supplemental Material 1. Survey Instrument
A 2024 cross-sectional survey of 2,389 university stakeholders (students and employees) on perspectives campus-wide tobacco use and the campus tobacco policy.

The CRoME Lab, a community-based research team housed at the School of Pharmacy, is requesting your participation in this brief, 5-minute survey and petition regarding tobacco use and policy on campus. Twenty-five (25) respondents will be randomly selected to win a cute UW-Madison mug.

Q1 What is your primary affiliation with UW-Madison?
□ _Undergraduate student (1)
□ _Graduate or professional degree student (2)
□ _Non-degree student (university special student, guest auditor, or noncredit continuing education student) (3)
□ _Faculty or staff (4)
□ _Other, please specify: __________ (5)

Q2 Where do you live during the current school year?
□ _University housing (residence hall, residential learning community, or university apartment community) (1)
□ _Fraternity or sorority house (2)
□ _Off-campus residence (house, apartment, condo, etc.) (3)
□ _Other, please specify: __________ (4)

Answer If: What is your primary affiliation with UW-Madison? “Faculty or staff” is Selected
Q3 How long have you been working at UW-Madison in years?

____

Q4 Secondhand smoke refers to smoke that you may breathe in that comes from someone else using a tobacco product. On a scale of 1 to 10, with 1 meaning “not at all harmful” and 10 meaning “extremely harmful”, how harmful do you believe secondhand smoke is to one's health.
□ _Not at all harmful; 1 (1)
□ _2 (2)
□ _3 (3)
□ _4 (4)
□ _5 (5)
□ _6 (6)
□ _7 (7)
□ _8 (8)
□ _9 (9)
□ _Extremely harmful; 10 (10)

Q5 How often are you exposed to secondhand smoke while you are on the UW-Madison campus?
□ _Never (1)
□ _Sometimes (2)
□ _About half the time (3)
□ _Most of the time (4)
□ _Always (5)

Q6 Secondhand aerosols from vaping refers to aerosols that you may breathe in that comes from someone else using an electronic vaping device (electronic cigarette, vape pen, etc.). On

a scale of 1 to 10, with 1 meaning “not at all harmful” and 10 meaning “extremely harmful”, how harmful do you believe secondhand e-cigarette aerosols is to one's health.
☐ _Not at all harmful; 1 (1)
☐ _2 (2)
☐ _3 (3)
☐ _4 (4)
☐ _5 (5)
☐ _6 (6)
☐ _7 (7)
☐ _8 (8)
☐ _9 (9)
☐ _Extremely harmful; 10 (10)

Q7 How often are you exposed to secondhand aerosols from other people vaping while you are on the UW-Madison campus?
☐ _Never (1)
☐ _Sometimes (2)
☐ _About half the time (3)
☐ _Most of the time (4)
☐ _Always (5)

Q8 Please indicate your level of awareness of UW-Madison’s current policy on tobacco use.
☐ _Very unaware (1)
☐ _Somewhat unaware (2)
☐ _Neither aware nor unaware (3)
☐ _Somewhat aware (4)
☐ _Strongly aware (5)

---Page break –

Note: The only part of UW-Madison’s campus that has a 100% tobacco-free policy is the Health Sciences Campus No-Smoking Zone outlined in the map below.

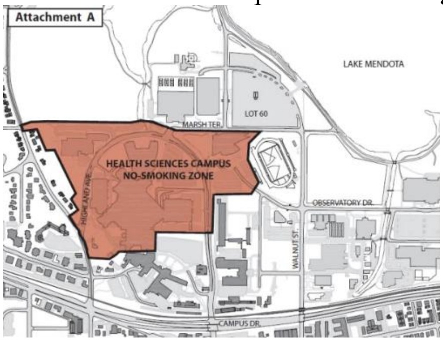

Q9 Please indicate your level of agreement with the following statement: “A tobacco- and vape-free campus policy at UW-Madison would encourage tobacco users to quit or reduce their consumption."
☐ _Strongly disagree (1)
☐ _Somewhat disagree (2)
☐ _Neither agree nor disagree (3)
☐ _Somewhat agree (4)
☐ _Strongly agree (5)

Q10 Please indicate your level of agreement with the following statement: “A tobacco- and vape-free policy at UW-Madison would promote a cleaner, greener, and healthier campus.”
☐ _Strongly disagree (1)

 _Somewhat disagree (2)
 _Neither agree nor disagree (3)
 _Somewhat agree (4)
 _Strongly agree (5)

Q11 Please indicate your level of support for a 100% tobacco-free campus policy for the entire UW-Madison campus.
 _Strongly oppose (1)
 _Somewhat oppose (2)
 _Neither oppose nor support (3)
 _Somewhat support (4)
 _Strongly support (5)

Q12 During the past 30 days, have you used any kind of tobacco product or electronic vaping product?
 _Yes (1)
 _No (2)

If “Yes” to Q12:
Q13 During the past 30 days, which of the following tobacco products and/or electronic vaping products have you used? (check all that apply)
 _Cigarettes (1)
 _Other tobacco products such as cigars, little cigars, cigarillos, pipes or hookah (2)
 _Smokeless tobacco products such as chewing tobacco, snuff, snus, dip, orbs, sticks, or strips (4)
 _Electronic vaping products, such as e-cigarettes (e-cigs), vape pens, hookah pens, or e-pipes (3)
 _Oral nicotine pouches (4)
 _Other, please specify: ____________ (5)

If “Yes” to Q12:
Q14 Please indicate when you first tried a tobacco product.
 _When you were under 18 years old (1)
 _18 to 20 years old (2)
 _When you were 21 years or older (3)
 _Other, please specify: _________________ (4)

If “Yes” to Q12:
Q15 Please indicate what best describes your frequency of use for the tobacco product(s) you selected above during your time at UW-Madison
 _Your use increased during your time at UW-Madison
 _Your use remained the same during your time at UW-Madison
 _Your use decreased during your time at UW-Madison

Q16 Petition Statement: I support the restructuring of the UW-Madison tobacco/vape-free campus policy.
 _ I agree (1)
 _ I disagree (2)
 _ I prefer not to answer (3)

If “I agree” for Q16:
Q17 Provide a statement of why you support the restructuring of the UW-Madison tobacco policy.

____

Q18 What other comments and questions do you have regarding the UW-Madison tobacco policy?

____

Provide your email address to be entered into the raffle for a UW-Madison-themed gift (Bucky Badger mug, UW-Madison printed tote bag, a UW-Madison baseball cap, or a UW-Madison-themed red umbrella)

Q19 Are you interested in working with us to pursue the 100% tobacco-free campus policy at UW-Madison?

- Yes, I am interested in helping out, and you can contact me at my email address above.
- No, I am not interested in helping.

Supplemental Material 2: Forrest Plot on Tobacco Use

Results from a 2024 cross-sectional survey of 2,389 university stakeholders (students and employees) on perspectives campus-wide tobacco use and the campus tobacco policy.

	OR (95% CI)	P-value
Student		0.0003
Yes	1 (Ref)	
No	0.53 (0.37-0.74)	
Year of affliated with UW-Madison		0.0066
0-1	1 (Ref)	
2-5	1.18 (0.85-1.63)	
>5	0.60 (0.39-0.91)	
Petition for Tobacco Policy Change		0.0013
Agree	1 (Ref)	
Disagree	2.47 (1.48-4.12)	
Prefer not to answer	1.23 (0.79-1.92)	
Second hand smoke harmful	0.69 (0.59-0.81)	<.0001
Exposed to SHA degree	1.34 (1.10-1.62)	0.0029
Awareness of current tobacco policy	1.19 (1.06-1.32)	0.0021
Agree on health	0.83 (0.71-0.97)	0.0192
Support tobacco-free	0.64 (0.54-0.78)	<.0001

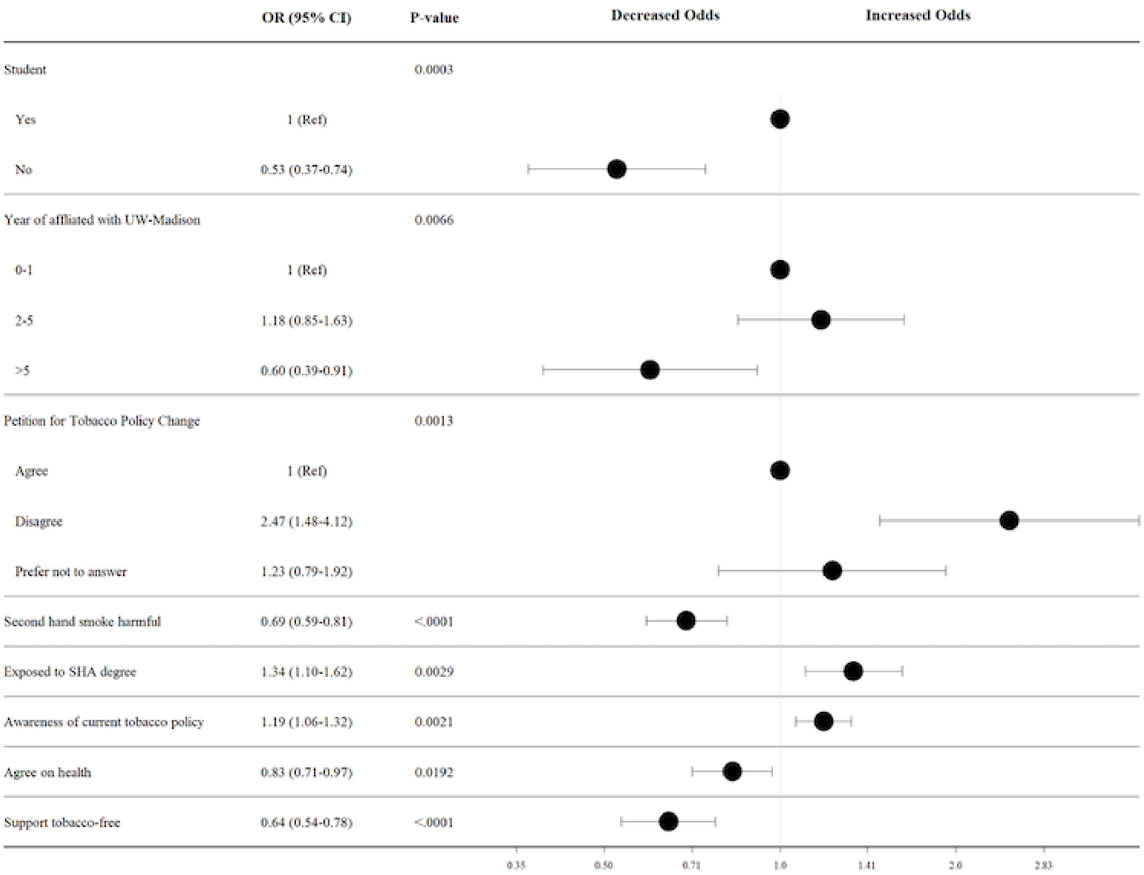

Supplemental Material 3: Forrest Plot on Support of a 100% Tobacco-free Campus Policy

Results from a 2024 cross-sectional survey of 2,389 university stakeholders (students and employees) on perspectives campus-wide tobacco use and the campus tobacco policy.

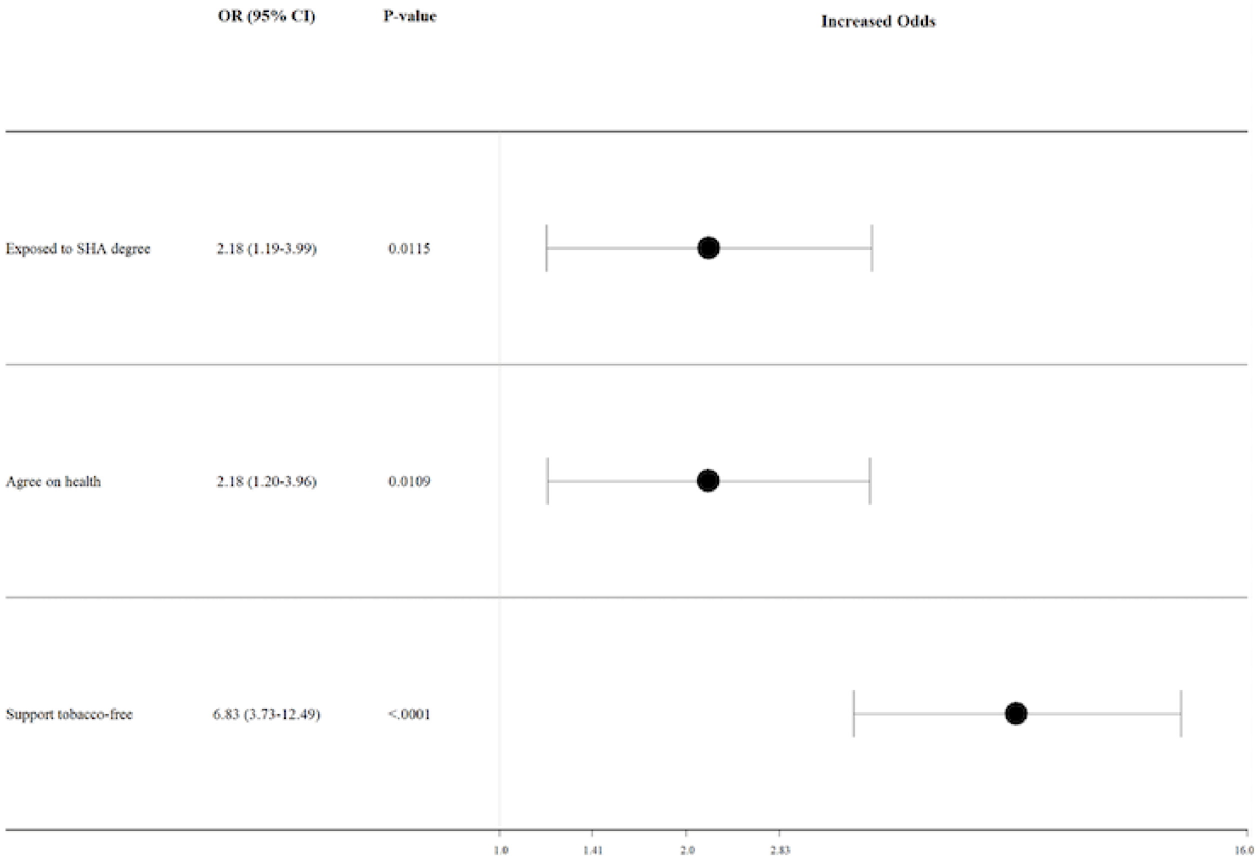

Supplemental Material 4: Themes and Verbatim Quotes for Support of a 100% Tobacco-free Policy

Results from a 2024 cross-sectional survey of 2,389 university stakeholders (students and employees) on perspectives campus-wide tobacco use and the campus tobacco policy.

Theme	Example Quote
Harmful Nature of Tobacco-containing Products	"Tobacco use is harmful to those who use it as well as those voluntarily or involuntarily in close proximity."
	"Tobacco is harmful to human health [and] promotions [of] a tobacco-free campus will incentivize individuals to quit smoking/vaping."
	"I believe that tobacco/vaping is harmful and should be banned. Since the university has the power to enforce this (compared to a nationwide ordinance), I think they should go for it."
Promotion of a Cleaner Campus Environment	"The air on campus should be safe for everyone to breathe."
	"More welcoming campus with cleaner air. Helps avoid unwanted exposure, especially for those with severe allergies and/or lung disease."
	"Tobacco and smoking are antiquated, severely harmful to health, and bad for the environment. I support UW-Madison taking a progressive step in eliminating tobacco use on campus for the health of our community and environment."
Limits Secondhand Smoke on Campus	"This is a harmful product, and smoking and vaping are harmful to people other than the smoker/vaper--innocent people who should not be subjected to someone else poisoning the air they breathe. There have been arguments in regard to vehicle emissions being dangerous, but there is a trade-off in that vehicles serve an important purpose. Smoking only harms people, and it is harmful to innocent bystanders. The paraphernalia from smoking/vaping/chewing, etc. are generally discarded by throwing them on the ground or out car windows, so the rest of us have to deal with smokers' trash as well. It is a terrible thing to allow people to do to other people."
	"Secondhand smoke is harmful to everyone's health, and it isn't fair for those who abstain from smoking to be exposed to it in the public places on campus."
	"Secondhand smoke is something I can't consent to, and yet it does negatively impact my health. Thus, someone's ability to choose to smoke does not, as far as I'm aware, outweigh my decision to not smoke or inhale the byproducts of another's decision."

Supplemental Material 5: Example Comments and Questions About the New Tobacco Campus Policy

Results from a 2024 cross-sectional survey of 2,389 university stakeholders (students and employees) on perspectives campus-wide tobacco use and the campus tobacco policy.

Theme	Example Quote
Tobacco Cessation Resources for Current Tobacco Users	"I think having a tobacco-free campus would be amazing! However, most people that use tobacco products can't just stop easily. So, along with new policies I think introducing new quitting resources and ad campaigns about quitting would be more beneficial!! Lots of people want to quit, but they need caring and understanding support to do so."
	"If there will be a change towards a 100% tobacco free campus, there should be help and support for tobacco addicts to quit, considering it is very addictive and restrictions don't always help people quit."
	"What types of programs would be offered to make a tobacco/vaping-free campus attainable for those that currently partake in these things? Support groups for quitting, etc."
Questions on Policy Implementation	"How will this be implemented? There are city streets, businesses, and apartments across campus, so how will the University ensure that tobacco use isn't happening around those areas by city members? What are the consequences if someone is using tobacco? Are those consequences different if it's a student/staff vs. a city member?"
	"I like the idea of a tobacco free campus, but I don't think it will be easy to implement especially with how big the campus is. People will find a way to smoke."
	"What would be the timeline for implementation?"
Questions on Policy Enforcement	"I don't think the policy is enforced. People will always try to disobey the policy, but it is only as effective as the enforcement of the policy."
	"Enforcement is the big question on my mind; I don't think there's a good way to enforce this. Fines on possibly marginalized communities are not great, and I really really really don't want to increase police presence on campus because criminalizing smoking is worse than allowing it."
	"The proposed policy is great, but a policy or law is only effective if enforced. What are the consequences of violations? Who enforces the policy?"

© 2025 Abraham O. et al.
